# Optimization of Automated Radiosynthesis of Gallium-68-Labeled PSMA11 with Two [^68^Ge]Ge/[^68^Ga]Ga Generators: Fractional Elution or Prepurification?

**DOI:** 10.3390/ph16111544

**Published:** 2023-11-01

**Authors:** Flore Durieux, Bérengère Dekyndt, Jean-François Legrand, Antoine Rogeau, Emmanuel Malek, Franck Semah, Pascal Odou

**Affiliations:** 1 Institute of Pharmacy, CHU Lille, F-59000 Lille, France; berengere.dekyndt@chu-lille.fr (B.D.); jeanfrancois.legrand@chu-lille.fr (J.-F.L.); pascal.odou@chu-lille.fr (P.O.); 2 Nuclear Medicine Department, CHU Lille, F-59000 Lille, France; antoinerogeau2109@gmail.com (A.R.); franck.semah@chu-lille.fr (F.S.); 3 EA 7365-GRITA—Groupe de Recherche sur les formes Injectables et les Technologies Associées, University of Lille, F-59000 Lille, France; 4 Radiopharmacy Unit—Institute of Pharmacy, CH Valenciennes, F-59300 Valenciennes, France; malek-e@ch-valenciennes.fr; 5 Inserm U1171, University of Lille, F-59000 Lille, France

**Keywords:** radiopharmacy, [^68^Ga]Ga-PSMA-11, double elution

## Abstract

Prostate cancer is one of the most common forms of cancer in men. An imaging technique for its diagnosis is [^68^Ga]-prostate-specific membrane antigen ([^68^Ga]Ga-PSMA-11) positron emission tomography (PET). To address the increasing demand for [^68^Ga]-labeled peptides and reduce the cost of radiosynthesis, it is therefore necessary to optimize the elution process of [^68^Ge]Ge/[^68^Ga]Ga generators. This study aims to identify the most effective approach for optimizing radiosynthesis using double elution in parallel of two [^68^Ge]Ge/[^68^Ga]Ga generators. Two methods have been tested: one using prepurification, and the other using fractionated elution. Five synthesis sequences were conducted using each method. The mean labeling yields for double elution with prepurification were 45.8 ± 29.4 (mean ± standard deviation) and none met the required criteria. The mean labeling yields for the fractionated double elution were 97.5 ± 1.9 (mean ± standard deviation) meeting the criteria, significantly superior to the prepurification method (*p* = 0.012), and similar to those of simple elution. This study showed that fractionated double elution from [^68^Ge]Ge/[^68^Ga]Ga generators produced a significantly higher labeling yield than double elution with prepurification, resulting in a larger activity recovered via radiosynthesis, thereby allowing more diagnostic tests to be performed.

## 1. Introduction

Prostate cancer is the 4th most common form of cancer in Europe and the most common in men [1]. According to the European Society for Medical Oncology (ESMO) guidelines, the diagnosis of high-risk forms relies on imaging techniques including magnetic resonance imaging (MRI), [^18^F]-fluorocholine positron emission tomography (PET), and [^68^Ga]-prostate-specific membrane antigen ([^68^Ga]Ga-PSMA-11) PET [2]. In France, [^68^Ga]Ga-PSMA-11 is a radiopharmaceutical drug available through compassionate access for patients with biologically recurrent prostate cancer, defined as a re-increase in serum prostate specific antigen (PSA) concentration [3]. This radiopharmaceutical drug has high sensitivity and excellent specificity for the diagnosis of prostate cancer and is increasingly used in routine practice [4].

The PSMA (Prostate Specific Membrane Antigen), is a transmembrane protein expressed by the epithelial cells of the prostate. In high-grade prostate cancer, PSMA is translocated to the luminal surface of the ducts and is overexpressed. When a ligand binds to the PSMA, it is internalized in the cell. This property provides an interesting target for both diagnosis and therapy [5].

Gallium-68 (t_1/2_ = 68 min) decays by positron emission (89%) and electron capture (11%). It has three oxidation states, +I, +II, +III. To achieve optimal radiolabeling, it must be in its +III state. This radiolabeling process is highly sensitive to pH: a too basic pH leads to the formation of precipitates and insoluble species, and a too-acidic pH leads to the formation of unstable species.

**Figure 1:**
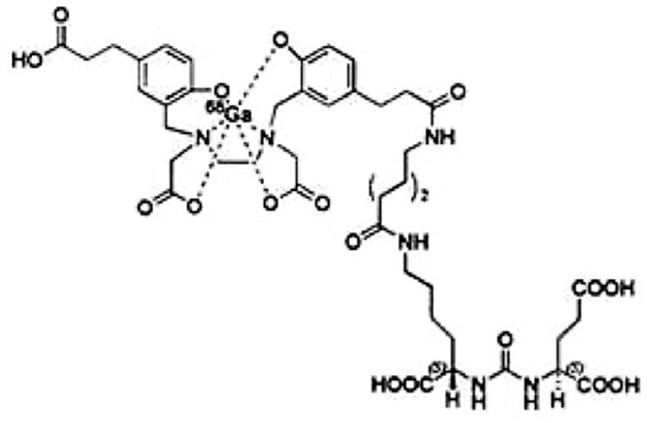
Chemicals structure of [^68^Ga]Ga-PSMA-11 (FDA).

This pH constraint necessitates the use of chelators such as HBED-CC, a component of PSMA-11. It enables ^68^Ga to be complexed in a stable manner. PSMA-11 also includes a Lys-CO-Glu bonding unit for binding to PSMA. These two parts are linked by a 6-aminohexanoic acid, which provides sufficient separation to prevent interference with each other [6,7] (Figure 1).

Gallium-68 can be obtained via cyclotron production with a [^68^Zn]Zn target solution or from elution of a germanium 68/gallium 68 ([^68^Ge]Ge/[^68^Ga]Ga) generator. These generators can be eluted on site, facilitating the production of radiolabeled PSMA without the constraints of industrial-scale production. This can be performed several times a day, and up to one year due to the ^68^Ge half-life of 271 days. However, they come with limitations which are their cost and a limited activity (only 1850 MBq capacity generator available in France). Due to the short half-life of ^68^Ga (68 min), the dosage of [^68^Ga]Ga-PSMA-11 (2 MBq/kg), and the time between injections (25 min), not many patients can be injected per elution. To address the growing demand for [^68^Ga]Ga-PSMA-11 and reduce the cost of the radiopharmaceutical per patient, the optimization of radiosynthesis has been implemented. Two potential options include eluting a generator calibrated with a higher activity or simultaneously eluting two generators in parallel [8].

In the present study, we investigate the radiosynthesis optimization of [^68^Ga]Ga-PSMA-11 based on the double elution in parallel of two [^68^Ge]Ge/[^68^Ga]Ga generators with calibration dates six months apart. This double elution is expected to increase the activity of radiosynthesis and ensure a more consistent supply of [^68^Ga]Ga-PSMA-11, thereby enhancing the continuity of PET exams over time.

Our objective was to compare two methods in order to determine the most suitable, i.e., the one that guarantees the best synthesis yield, in accordance with the Good Manufacturing Practices for Preparations:

- Double elution with prepurification to concentrate the gallium 68;
- Double fractional elution to extract the most concentrated fraction in activity of the two eluates.

## 2. Results

A total of 10 radiosynthesis sequences were performed: 5 with prepurification and 5 with fractionated elution. All yields (elution and labeling) were measured at the time of elution.

### 2.1. Double Elution with Prepurification

The labeling yields of the elution with prepurification were between 6 and 74% and therefore did not comply with the expected yields. Furthermore, the radiolabeling success was inconsistent (mean of labeling yield: 45.8 ± 29.4%). For the second radiosynthesis, the quality control was not compliant with the HPLC (6.7% of impurities). For the third radiosynthesis, the quality controls were not compliant with a TLC-assessed RCP = 71.8%. For this radiosynthesis, HPLC was not performed because the TLC was already not compliant. The elution yields were compliant to specifications (63.8 ± 4.8%). Visual examination, pH values, and endotoxins levels measured at the end of radiosynthesis were constantly compliant. The radiosynthesis results are detailed in Table 1.

### 2.2. Double Fractional Elution

The labeling yields and quality control requirements were met for all sequences. The labeling yields were between 94% and 99%, and the elution yields were compliant to specifications (59.4 ± 2.2%). Visual examination, pH values and endotoxins levels measured at the end of radiosynthesis were constantly compliant. The radiosynthesis results are detailed in Table 2.

### 2.3. Statistical Tests

There was no significant difference between the elution yields of both methods (*p* = 0.151), but there was a significant difference in labeling yields between the prepurification and the fractionated elution methods (*p* = 0.012), in favor of the latter. Regarding overall radiosynthesis yields, the difference was also significant (*p* = 0.008).

## 3. Discussion

In the present study, we compare the radiosynthesis and quality control results of two different preparation methods for [^68^Ga]Ga-PSMA-11. We demonstrate that double elution with prepurification led to insufficient and greatly varying radiolabeling yield of [^68^Ga]Ga-PSMA-11, whereas fractionated double elution resulted in consistent and higher labeling/overall yield.

There are several potential explanations for the results obtained with elution with prepurification. One possibility is related to pH issues. As previously mentioned, pH plays a critical role in the radiosynthesis of [^68^Ga]Ga-PSMA-11, and a too-acidic pH can result in the protonation of the chelator. On the other hand, if the pH is too basic, it can lead to the formation of insoluble $Ga({OH)}_{3}$. In this method, 5 mL of an HCl/NaCl mixture were added to 3 mL of acetate buffer. In contrast, in the double fractionation elution method, 6 mL of ^68^Ga in HCl 0,1M is added to 1 mL of acetate buffer. Given the differences in the reaction medium, the pH may vary. Unfortunately, pH measurement at this step is not an “in-process” checkpoint leaving doubt regarding the role of pH in the synthesis failure [6,9].

Another hypothesis that could have been considered is the presence of impurities in the reaction vial. Metallic impurities may result from the degradation of the generator column or the purification process. Ferric impurities may compete with ^68^Ga, preventing its complexation with the chelator and thereby decreasing the yield. However, this hypothesis was effectively ruled out since simple elution (i.e., elution of one [^68^Ge]Ge/[^68^Ga]Ga generator for radiosynthesis) was performed and quality checks and yields met requirements (see Appendix A, Table A1) [7,10].

As a result of these findings, we began to reevaluate the overall preparation process. Interestingly, Reverchon et al. [11] demonstrated that the optimal conditions for the preparation of [^68^Ga]Ga-PSMA-11 might involve no purification and no heating, leading to a significant reduction in preparation time. Under these conditions, quality controls indicated an RCP of over 99% and a radiolabeled yield of over 99%. However, this approach presents its own challenges. Removing the purification step leads to a larger elution volume (10 mL), and requires a complete modification of the radiosynthesis process including adjustments to the acetate buffer volume, heating profile, and volume activity for labeling.

Finally, all fractional elution tests produced results within specifications. Fractional elution appears to be the method of choice for [^68^Ga]Ga-PSMA-11 radiosynthesis for several compelling reasons. Firstly, as mentioned earlier, fractional elution enables the removal of the eluate portion containing metallic impurities, while retaining the part with the highest activity for radiolabeling with nearly the same volume as simple elution. Although the elution yields may be slightly lower, the labeling yields are significantly higher. Secondly, the preparation process is simplified as the need for prepurification is eliminated, making it similar to simple elution, which exhibits high labeling yields. Lastly, the simplified preparation process not only saves time but also results in higher activity.

## 4. Materials and Methods

### 4.1. Materials Used for Radiosynthesis with Prepurification and Fractional Double Elution

For both methods, radiosynthesis was performed on MiniAIO^®^ synthesis module (Trasis^®^, Ans, Belgium) placed in high-energy shielded enclosure H700hotcell (Trasis^®^). The peptide radiolabeled was PSMA-11 from Iason GmbH^®^ (Graz, Austria). Sterile solution of gallium 68 was obtained with [^68^Ge]Ge/[^68^Ga]Ga generators (Galliapharm^®^, Eckert & Ziegler Radiopharma GmbH, Berlin, Germany). We used elution solvent 1 M hydrochloric acid from Eckert & Ziegler, an HLB cartridge, and NaCl 0.9%.

For double elution with prepurification, we used acetate buffer 1 M and absolute ethanol from Trasis^®^ (Ans, Belgium), cassette with prepurification (ref: S10886) from Trasis^®^, C18 prepurification cartridge Oasis (Waters^®^, Milford, CT, USA) and Suprapur^®^ NaCl/HCl 30% (Trasis^®^).

For double fractional elution, we used acetate buffer 0.7 M and absolute ethanol prepared extemporaneously and cassette without prepurification from Trasis^®^ (ref: S7577).

### 4.2. The Double Elution with Prepurification Method

**Figure 2:**
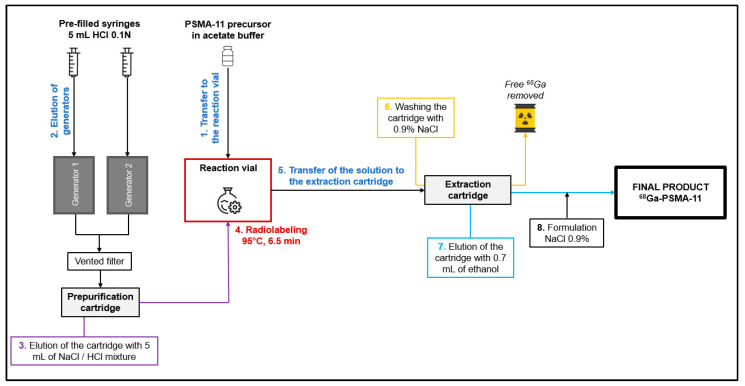
Overview of the double elution with prepurification.

A cassette with prepurification is placed on the MiniAIO^®^ synthesizer. An amount of 10 µg of PSMA-11 is reconstituted with 3 mL of sodium acetate buffer from Trasis^®^ and then transferred into the reaction vial (step 1 in Figure 2). The elution of the two [^68^Ge]Ge/[^68^Ga]Ga generators is performed in parallel, using 5 mL of HCl 1M (2 mL/min) each. A female Luer lug style T and a ventilated filter are positioned at the outlet of the two generators (step 2). ^68^Ga solution is transferred to the prepurification cartridge which is eluted with 5 mL of a Suprapur^®^ NaCl/HCl 30% mixture (step 3). The labeling reaction is performed at 95 ± 5 °C during 6.5 min (step 4). The solution is loaded onto an HLB cartridge to retain the [^68^Ga]Ga-PSMA-11. This cartridge is rinsed with 0.9% NaCl. The free ^68^Ga is evacuated in waste (steps 5 and 6). The cartridge is eluted with 0.7 mL of absolute ethanol, recovering the [^68^Ga]Ga-PSMA-11 (step 7). The solution is transferred to the final vial and, finally, formulated with 9.3 mL of 0.9% NaCl. Volume of the final product is 10 mL (step 8).

### 4.3. The Double Fractional Elution Method

**Figure 3:**
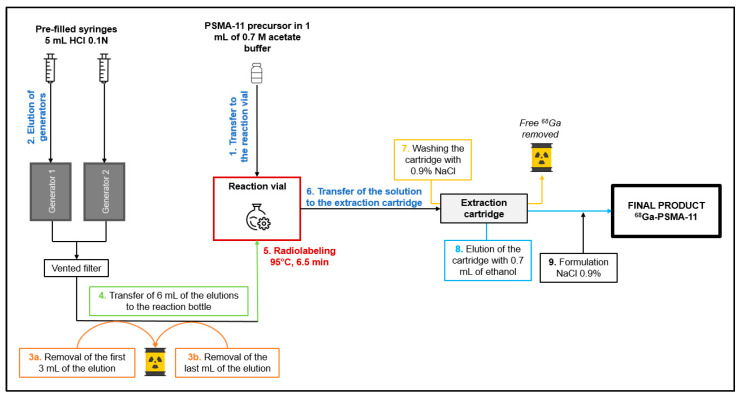
Overview of the double fractional elution.

**Figure 4:**
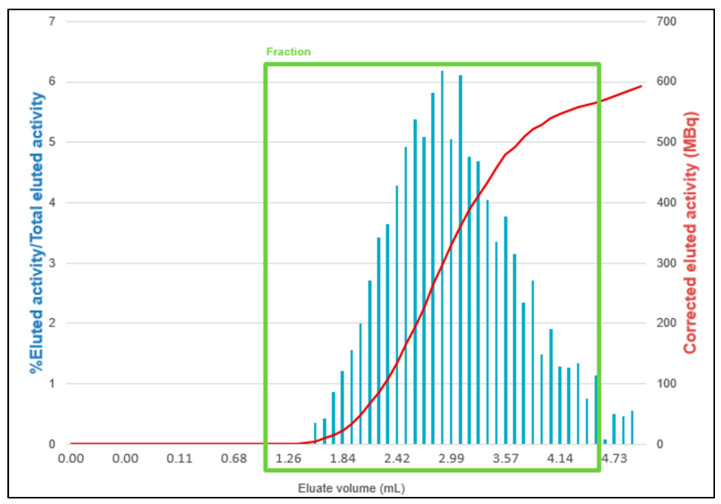
Profile of unfractionated ^68^Ga elutions. From Boukhlef et al. [12].

A cassette without prepurification is placed on the MiniAIO^®^ synthesizer. An amount of 10 μg of PSMA-11 is reconstituted with 1 mL of 0.7 M sodium acetate solution, prepared, and filtered in radiopharmacy under laminar flow (ISO 5 from the NF EN ISO 14644-1 standard), and then transferred into the reaction vial (step 1 in Figure 3). The elution of the two [^68^Ge]Ge/[^68^Ga]Ga generators is performed in parallel, using 5 mL of HCl 1M (2 mL/min) each. A T device and a ventilated filter are positioned at the outlet of the two generators (step 2). The first 3 mL, i.e., 1,5 mL for each generator and the last 1 mL, i.e., 0,5 mL for each generator, of the elution are eliminated in waste (steps 3a and 3b). These volumes were established according to a study [12] showing that fractionation of the elution from the [^68^Ge]Ge/[^68^Ga]Ga generators resulted in a higher activity volume. The following diagram from the study shows this result (Figure 4).

An amount of 6 mL of the elution, the most active fraction, is transferred to the reaction vial containing the peptide in solution in acetate buffer (step 4). The labeling reaction is performed at 95 ± 5 °C for 6.5 min (step 5). The solution is loaded onto a C18 cartridge (SEP-Pak) which retains the [^68^Ga]Ga-PSMA-11. The cartridge is rinsed with 0.9% NaCl (step 6). The cartridge is eluted with 0.7 mL of absolute ethanol removing the [^68^Ga]Ga-PSMA-11 transferred to the final vial. The final product is then diluted with 9.3 mL of 0.9% NaCl (steps 7 and 8).

### 4.4. The Acceptance Criteria

For each synthesis procedure, quality controls were carried out:

1. Visual examination of organoleptic character: the product must be clear and colorless.
2. pH measured with a pH strip must be between 4 and 8.
3. Radiochemical purity (RCP), measured via two methods: (1) High performance liquid chromatography (HPLC) with C18 column Waters^®^ 250 × 4.6 mm, flow: 1 mL/min, with solvent gradient Acetonitril/TFA 0.1% and water/TFA 0.1%. The programming is 00:00–3:00 min: 97% A—3% B; 06:00–9:00 min: 0% A—100% B and 12:00–15:00 97% A—3% B. The retention time is 3.7 min for free ^68^Ga and 8.3 min for [^68^Ga]Ga-PSMA-11. The addition of the stereoisomers is at least 95% of the total radioactivity due to 68Ga. (2) Thin-layer chromatography (TLC) with ITLC-SG plates, where the retention factor of [^68^Ga]Ga-PSMA-11 is 0.8–1, that of 68Ga 3+ is 0–0.1, and that of the ^68^Ga colloid is 0.7. No less than 95% of the total radioactivity is due to [^68^Ga]Ga-PSMA-11.
4. Bacterial endotoxins, measured via a kinetic and chromogenic LAL method (Endosafe^®^, Charles River, Wilmington, NC, USA): the preparation is diluted to the 10th and deposited on PTS-10 cassettes with a sensitivity of 0.01EU/ML-PTS2001. The expected result is a bacterial endotoxin content lower than 15.1 IU/mL.
5. Radiolabeling yield: measured with an ISOMED2010^®^ dose calibrator, which is expected to be higher than 80%.
6. Elution yields were calculated. The manufacturer specification of elution yield is >62%, but the prepurification/fractioning steps tend to decrease this yield (about 10%), and therefore, we set the specification to an elution yield > 55%.

### 4.5. Statistical Tests

A Mann–Whitney test was performed to compare elution and labeling yields as well as the overall radiosynthesis yields (multiplication of the elution and labeling yields) of the two methods.

## 5. Conclusions

The literature concerning fractional elution for PSMA-11 radiolabeling remains limited, and our study demonstrates that this method applied on a [^68^Ge]Ge/[^68^Ga]Ga generator can optimize radiosynthesis. Fractional elution facilitates a higher-activity product enabling a greater number of patients to benefit from [^68^Ga]Ga-PSMA-11 PET for diagnosis, while reducing the frequency of radiosynthesis per week. This optimization can help to plan for the anticipated huge increase in [^68^Ga]Ga-PSMA-11 PET-scans. Moreover, the double fractional elution approach could potentially be extended to the labeling of other peptides, such as edotreotide.

## Figures and Tables

**Table 1:** Results of double elution radiosynthesis with prepurification.

Radiosynthesis	1	2	3	4	5	
Yield						Mean ± SD
Elution yield (%)	62.7	71.7	64.5	59.4	60.7	63.8 ± 4.8
Labeling yield (%)	73.9	54.3	6.4	24.4	69.9	45.8 ± 29.4
Elution yield × Labeling yield (%)	46.3	38.9	4.1	14.5	42.4	29.2 ± 18.8
**Quality control**						
pH	6	6	6	6	5	5.8 ± 0.4
^68^Ga free rate in TLC (%)	1.7	4.5	28.2	0.1	1.0	7.1 ± 11.9
^68^Ga free rate in HPLC (%)	3.3	6.7	-	4.6	4.6	4.8 ± 1.4
Radiochemical purity (%)	96.7	93.3	71.8	95.4	95.4	90.5 ± 10.6
Endotoxins (UI/mL)	<15.1	<15.1	<15.1	<15.1	<15.1	

**Table 2:** Results of fractionated double elution radiosynthesis.

Radiosynthesis	6	7	8	9	10	
Yield						Mean ± SD
Elution yield (%)	61.2	56.1	59.5	61.6	58.8	59.4 ± 2.2
Labeling yield (%)	97.1	98.1	94.5	99.0	99.0	97.5 ± 1.9
Elution yield × Labeling yield (%)	59.4	55.0	56.2	61.0	58.2	58 ± 2.4
**Quality control**						
pH	6	5.5	5	5	5	5.3 ± 0.4
^68^Ga free rate in TLC (%)	1.8	1.0	0.7	1.1	0.4	1 ± 0.5
^68^Ga free rate in HPLC (%)	3.6	3.3	4.2	3.1	4.2	3.7 ± 0.5
Radiochemical purity (%)	96.4	96.7	95.8	97.0	95.8	96.3 ± 0.5
Endotoxins (UI/mL)	<15.1	<15.1	<15.1	<15.1	<15.1	

## Data Availability

All data generated or analyzed during this study are included in this published article.

## Appendix A

**Table A1:** Results of simple elution radiosynthesis.

Radiosynthesis	11	12	13	14	15	
Yield						Mean ± SD
Elution yield (%)	62.9	65.5	65.2	65.7	68.0	65.5 ± 1.8
Labeling yield (%)	99.2	95.7	94.0	94.5	95.8	95.8 ± 2.0
Elution yield × Labeling yield (%)	62.4	62.7	61.3	62.1	65.1	62.7 ± 1.5
**Quality Control**						
pH	6	5	6	6	6	5.8 ± 0.4
^68^Ga free rate in TLC (%)	2.5	0.3	1.4	2.0	2.7	1.8 ± 1.0
^68^Ga free rate in HPLC (%)	2.0	1.5	3.1	1.8	2.6	2.2 ± 0.6
Radiochemical purity (%)	97.5	98.5	96.9	98.0	97.3	97.6 ± 0.6
Endotoxins (UI/mL)	<15.1	<15.1	<15.1	<15.1	<15.1	
